# Associations of Betatrophin/ANGPTL8 with Septic Dyslipidemia in Human Peritonitis: An Explorative Analysis

**DOI:** 10.3390/biomedicines10123151

**Published:** 2022-12-06

**Authors:** Paul Horn, Sascha Radtke, Uta Barbara Metzing, Ricardo Steidl, Christoph Sponholz, Oliver Sommerfeld, Johannes Roth, Ralf A. Claus, Andreas L. Birkenfeld, Utz Settmacher, Falk Rauchfuß, Christian von Loeffelholz

**Affiliations:** 1 Department of Internal Medicine IV, Gastroenterology, Hepatology and Infectious Diseases, Jena University Hospital, 07747 Jena, Germany; 2 Department of Anaesthesiology and Intensive Care, Jena University Hospital, Friedrich Schiller University, Am Klinikum 1, 07747 Jena, Germany; 3 Department of Trauma, Hand and Reconstructive Surgery, Jena University Hospital, Friedrich Schiller University, Am Klinikum 1, 07747 Jena, Germany; 4 Department of Diabetology Endocrinology and Nephrology, University Hospital Tübingen, Eberhard Karls University Tübingen, 72076 Tübingen, Germany; 5 Department of Therapy of Diabetes, Institute of Diabetes Research and Metabolic Diseases in the Helmholtz Center Munich, Eberhard Karls University Tübingen, 72076 Tübingen, Germany; 6 Division of Diabetes and Nutritional Sciences, Rayne Institute, King’s College London, London SE5 9RJ, UK; 7 Department of General, Visceral and Vascular Surgery, Jena University Hospital, Am Klinikum 1, 07747 Jena, Germany

**Keywords:** adipose tissue, type 2 diabetes, hypertriacylglyceridemia, insulin sensitivity, very low-density lipoprotein

## Abstract

Sepsis is defined by life-threatening organ dysfunction mediated by the host’s response to infection. This can result in septic dyslipidemia, which is involved in the neutralization of pathogen-related lipids. Knowledge of the regulatory mechanisms of septic dyslipidemia is incomplete. The cytokine betatrophin/Angiopoietin-like protein 8 (ANGPTL8) plays a role in the regulation of triacylglyceride metabolism, though its function in septic dyslipidemia remains unknown. Sixty-six patients were enrolled in a cross-sectional study. Circulating concentrations and adipose tissue (AT) mRNA expression of betatrophin/ANGPTL8 were studied in patients suffering from peritoneal sepsis. Insulin-resistant individuals and subjects without metabolic derangement/systemic inflammation were enrolled as controls. All underwent open abdominal surgery. Circulating betatrophin/ANGPTL8 was analyzed by an enzyme-linked immunosorbent assay and AT mRNA expression levels were assessed by real-time PCR. Standard laboratory analyses including lipid electrophoresis were evaluated. Sepsis patients showed pronounced septic dyslipidemia (*p* < 0.05 for all major lipid classes). Despite comparable betatrophin/ANGPTL8 mRNA expression in AT (*p* = 0.24), we found significantly increased circulating betatrophin/ANGPTL8 with septic dyslipidemia (*p* = 0.009). Expression levels of betatrophin/ANGPTL8 in AT correlated with circulating concentrations in both control groups (r = 0.61; *p* = 0.008 and r = 0.43; *p* = 0.034), while this association was undetectable in sepsis. After stratification, betatrophin/ANGPTL8 remained associated with hypertriacylglyceridemia (*p* < 0.05).

## 1. Introduction

Sepsis is characterized by organ dysfunction and initiated by the dysregulated host response to infection [1,2]. Beyond other adaptive mechanisms, impaired mitochondrial function and extensive metabolic reprogramming are recognized hallmarks in the pathogenesis of septic shock [3]. Alterations of glucose metabolism and insulin sensitivity have been comprehensively studied under proinflammatory conditions, while knowledge on the role of lipid metabolism in sepsis remains scarce. Using gold standard methods, it was shown that by interference with insulin signaling, systemic inflammation may not exclusively modify glucose metabolism, but it also affects adipose tissue (AT) lipolysis, resulting in a rise of circulating free fatty acids (FFA) [4]. Along with lipopolysaccharides (LPS) from bacterial cell walls and further complementary factors, this can lead to inflammation-related dyslipidemia and specifically promote septic hypertriaclylglyceridemia [5,6]. Current research relates dyslipidemia to the severity of organ dysfunction and prognosis in septic humans [7,8,9,10,11]. Otherwise, potential mediators of reprogrammed lipid metabolism due to major systemic inflammation are widely unknown.

The cytokine betatrophin, also known as Angiopoietin-like protein 8 (ANGPTL8), was originally thought to be related to insulin resistance and type 2 diabetes mellitus (T2D) [12,13]. However, current experimental and clinical evidence rather suggests a primary role in the regulation of lipid and particularly triacylglyceride (TAG) metabolism [14,15,16]. Furthermore, in one study, the cytokine was positively correlated with systemic inflammation [17]. However, the potential role of betatrophin/ANGPTL8 in septic dyslipidemia is unknown as yet. Therefore, we aimed at providing a first explorative characterization of this cytokine under such conditions in humans for a better understanding as a potential marker or mediator of deranged lipid metabolism during the early host response.

## 2. Materials and Methods

### 2.1. Study Design

This cross-sectional study evaluates the association of the cytokine betatrophin/ANGPTL8 with selected parameters of lipid metabolism in septic patients. For this purpose, we investigated blood and AT samples from a previous human study, which only included patients undergoing laparotomy [18]. The major lipid classes total cholesterol (TC), low-density lipoprotein (LDL), high-density lipoprotein (HDL), FFA, and circulating TAG were analyzed as surrogates of disease-related metabolic reprogramming.

We further included surgical patients suffering from insulin resistance and low-grade inflammation yet free from infection or sepsis as one control group, since betatrophin/ANGPTL8 in circulation and tissue expression rates can be altered under such conditions [19,20]. Subjects with absent sepsis and insulin resistance but undergoing therapeutic abdominal surgery were included as a second control group.

Baseline data from standard laboratory analyses and clinical measurements were collected. Blood and visceral adipose tissue (VAT) were sampled. Circulating concentrations and gene expression levels of betatrophin/ANGPTL8 in VAT were analyzed.

### 2.2. Subjects and Ethics

This study was based on a secondary analysis of previously harvested samples [18]. The protocol was approved by the faculty’s ethics review board of the Jena University Hospital (3247-09/11), and all enrolled subjects, or their legal representatives, gave written informed consent. Patients were aged 18 years or older and had not undergone other surgical interventions in the five days prior to enrollment. Septic patients met the criteria of the German Sepsis Society [21]. Patients were included in the insulin resistance group if they had known T2D using antidiabetic therapy or met the criteria of the American Diabetes Association and/or of the National Cholesterol Education Adult Treatment Panel III for metabolic syndrome [22,23]. Antidiabetic medication was routinely discontinued before elective surgery in T2D patients according to national treatment standards. Subjects enrolled in the control group did not match the above-mentioned criteria for the diagnosis of insulin resistance or sepsis. General exclusion criteria were long-term immune-suppressive treatment, history of organ transplantation, pre-existing chronic kidney disease or kidney failure with essential hemodialysis, chemotherapy within the last two months, drug or alcohol abuse (defined as a daily alcohol intake of more than 20 g for females and 40 g for males), active rheumatoid inflammatory disease, and known liver cirrhosis.

### 2.3. Blood and Tissue Sampling

Peripheral blood was taken in the morning before laparotomy from all subjects of the control groups after an overnight starvation period. Due to logistic reasons arising from emergency surgery in patients with sepsis, blood from these subjects was obtained on the morning of the day after surgery after an overnight fasting period of at least eight hours. Blood samples remained on ice until centrifugation. The serum was prepared by centrifugation at 3000× *g* for 10 min at 4 °C and then stored at −80 °C. All clinical laboratory parameters including FFA were measured in certified laboratories of our University Hospital according to established clinical standards. Whole-body insulin resistance was assessed by calculation of the homeostasis-model assessment of insulin resistance (HOMA-IR) [13]. VAT samples were taken right after midline incision and preparation, immediately snap-frozen in liquid nitrogen, and stored at −80 °C until further processing.

### 2.4. Quantification of Circulating Betatrophin/ANGPTL8

Levels of circulating betatrophin were measured from human serum by enzyme-linked immunosorbent assay (ELISA) using the Human Betatrophin/ANGPTL8 ELISA kit (BioVendor, Karasek, Czech Republic). We performed the ELISA according to the manufacturer’s instructions, measured all samples in duplicates, and calculated the means.

### 2.5. Betatrophin/ANGPTL8 Expression in VAT

Extraction and analyses were performed by using established protocols [13,18]. Quantitative real-time (qRT) PCR was used for mRNA analysis. Briefly, we extracted the total RNA using the RNeasy Lipid Tissue Mini Kit (QIAGEN, Hilden, Germany) according to the manufacturer’s instructions. The total RNA concentration was measured by spectrophotometry with the NanoDrop 1000 (NanoDrop Products/Thermo Scientific, Wilmington, DE, USA), and integrity was tested with automated electrophoresis with the Experion Automated Electrophoresis System (Bio-Rad Laboratories, Hercules, CA, USA) and found to conform with high-quality requirements. Extracted RNA was transcribed into cDNA with the Revert Aid First Strand cDNA Synthesis Kit (Fermentas, Waltham, MA, USA). PCR reactions were performed on Rotor-Gene Q (QIAGEN, Hilden, Germany) in a total reaction volume of 20 μL with Brilliant II SYBR Green qPCR Master Mix (Stratagene, La Jolla, CA, USA) and forward and reverse primers (Biomers, Ulm, Germany). Relative expression for each primer was interpolated from standard curves. After housekeeping gene analysis, the means of relative expression levels of β actin (ACTB), glyceraldehyde 3-phosphate dehydrogenase (GAPDH), hypoxanthine phosphoribosyltransferase 1 (HPRT), and hydroxymethylbilane synthase (HMBS) were calculated and used as normalization factors. Primer sequences used for analysis were as follows: f: ctgtcccgtagcaccttctg, r: cagaaggtgctacgggacag for ANGPTL8/betatrophin; for ACTB, f: ggcatgggtcagaaggatt, r: aggtgtggtgccagattttc; for GAPDH, f: ctctgctcctcctgttcgac, r: caatacgaccaaatccgttgac; for HPRT, f: cctggcgtcgtgattagtgat, r: agacgttcagtcctgtccataa; and for HMBS, f: atgtctggtaacggcaatgc, r: cgtctgtatgcgagcaagc; reverse primers were exonspanning, respectively [18].

### 2.6. Statistical Analysis

All statistical analyses were performed in SPSS 22.0 (SPSS Inc., Armonk, NY, USA), R version 4.0.5 (The R Foundation for Statistical Computing), and the rstatistix package. Graphs were drawn using RStudio with ggpubr and ggplot2 packages.

Data are shown as means ± SD, if not stated otherwise. Boxes in all boxplots span from the 25th–75th percentiles, whiskers indicate the minimum and maximum values inside the 1.5-times interquartile range above and below the first and third quartiles, and outliers are depicted as dots. The normal distribution was tested with the Shapiro–Wilks test. To test for the homogeneity of variance, we used the Levene procedure. Depending on the data distribution, we used the following statistical procedures: One-way analysis of variance (ANOVA), Welch’s test, or the Kruskal–Wallis test with post hoc Bonferroni-Holm or Dunn-Holm correction, and for testing of ordinal or nominal data, we used the χ²-test. Correlation analysis was performed using the Spearman rank correlation coefficient. An alternative hypothesis was accepted if two-sided *p* < 0.05.

## 3. Results

### 3.1. Patient characteristics

Baseline data of enrolled patients are given in Table 1, the indications for abdominal surgeries are provided in Supplementary Table S1, and clinical information on the group of sepsis subjects is provided in Supplementary Table S2.

Subjects meeting the criteria of sepsis showed elevated CRP and leukocyte counts (*p* < 0.05, respectively). Moreover, creatinine and γGT were increased, while thromboplastin time was reduced (*p* < 0.05 for all), suggesting sepsis-related organ dysfunction. Septic patients had reduced TC, LDL, and HDL, while at the same time showing increased TAG, verifying the presence of septic dyslipidemia (*p* < 0.05 for all).

Pronounced differences were observed regarding the parameters of age and BMI between septic patients and controls with absent insulin resistance/inflammation (*p* < 0.05, respectively). Otherwise, those parameters were well comparable in the insulin resistance and sepsis group. HbA1c as a representative indicator of chronic metabolic derangement showed equivalent values in septic patients and subjects with absent insulin resistance/inflammation, while it was elevated in the insulin resistance group (*p* < 0.05). The presence of low-grade inflammation in the latter group was verified by slightly elevated CRP as compared to metabolically healthy controls.

### 3.2. Betatrophin/ANGPTL8 under Conditions of Major Inflammation vs. Isolated Metabolic Dysregulation

**Figure 1:**
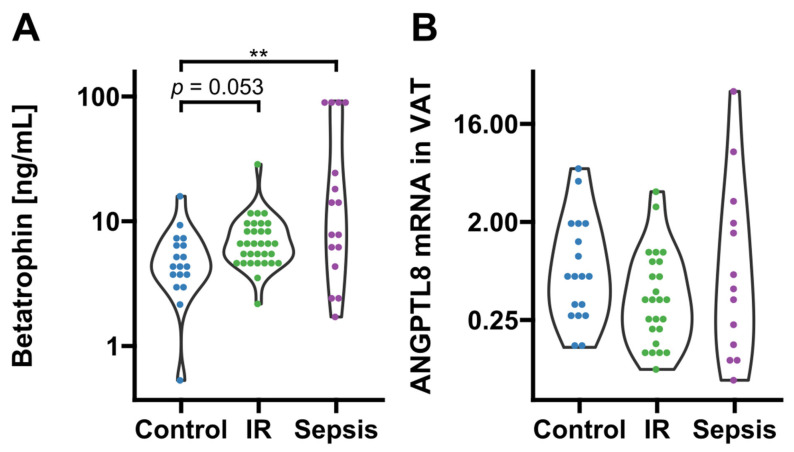
Circulating levels and adipose tissue expression of betatrophin/ANGPTL8. (**A**) Circulating betatrophin/ANGPTL8 was significantly increased in septic (*p* = 0.009 after post hoc correction, n = 16) and non-significantly increased in insulin-resistant subjects (*p* = 0.053 after post hoc correction, n = 32) as compared to metabolically healthy controls with absent inflammation (n = 18; overall significance according to Kruskal–Wallis *p* = 0.009). (**B**) Betatrophin/ANGPTL8 gene expression in VAT was comparable between groups (sepsis, n = 13; insulin resistance, n = 32, controls n = 18). IR, insulin resistance; VAT, visceral adipose tissue. Dots are colored according to group identities given on the x-axis. ** *p* < 0.01 in Kruskal-Wallis test and post-hoc Dunn correction.

**Figure 2:**
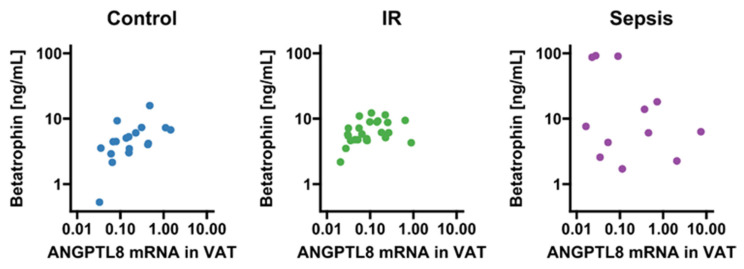
Correlation analysis of betatrophin/ANGPTL8 circulating levels and VAT mRNA expression. Correlations were observed in subjects free from inflammation and metabolic dysregulation (r = 0.61; *p* = 0.008) and in insulin-resistant patients (r = 0.43; *p* = 0.034), but not in the sepsis group (r = −0.32; *p* = 0.34). IR, insulin resistance; VAT, visceral adipose tissue. Dots are colored according to group identities given in panel titles.

Circulating betatrophin/ANGPTL8 was detectable in 66 subjects (sepsis: n = 16; insulin resistance: n = 32; no insulin resistance/no systemic inflammation: n = 18). Compared to conditions of absent metabolic dysregulation and systemic inflammation, values of circulating betatrophin/ANGPTL8 were significantly increased in sepsis and non-significantly increased in the insulin resistance group (*p* = 0.009 and *p* = 0.053, respectively; Figure 1A). Although betatrophin/ANGPTL8 expression in VAT remained grossly comparable between the three groups (*p* > 0.05; Figure 1B), we observed a correlation of mRNA expression with circulating levels exclusively in insulin-resistant patients and metabolically healthy subjects with absent systemic inflammation (*p* < 0.05, respectively; Figure 2).

Of note, we observed no associations of circulating betatrophin/ANGPLT8 with insulin resistance as estimated by HOMA-IR in any of the three subgroups (*p* > 0.05, respectively; see Supplementary Figure S1). We otherwise found a significant negative correlation with HbA1c in septic subjects, and a negative correlation of betatrophin/ANGPLT8 VAT mRNA expression with HbA1c in the insulin-resistant group (*p* < 0.05, respectively; see Supplementary Figure S1). Otherwise, after adjustments for multiple tests, the observed correlations no longer remained significant. In addition, decreased betatrophin/ANGPLT8 mRNA VAT expression was found in patients with chronic Metformin treatment (*p* < 0.05; Supplementary Figure S1).

### 3.3. Associations of Betatrophin/ANGPTL8 with Surrogate Parameters of Lipid Metabolism under Conditions of Septic Dyslipidemia

**Figure 3:**
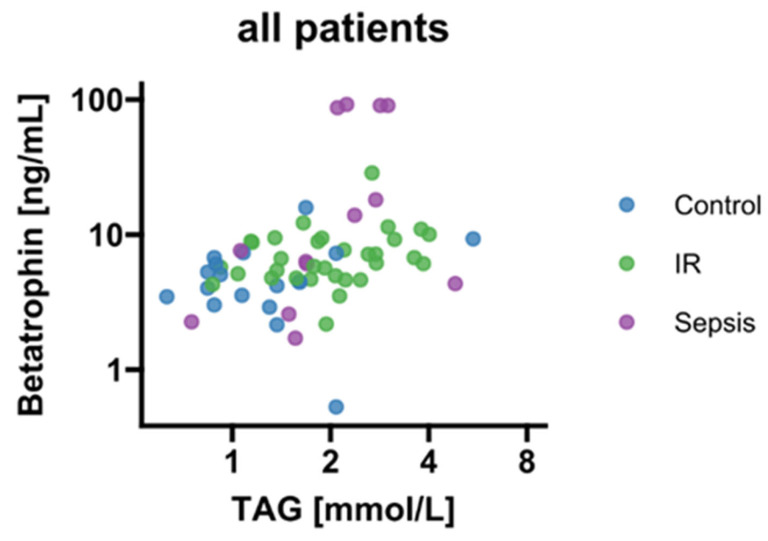
Correlation analysis of circulating betatrophin/ANGPTL8 and TAG (r = 0.46; *p* < 0.001). IR, insulin resistance; TAG, triacylglycerides.

**Figure 4:**
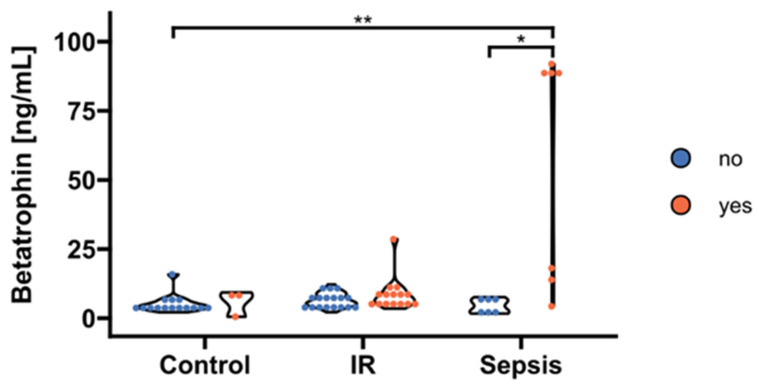
Stratification of circulating betatrophin/ANGPTL8 according to hypertriacylglyceridemia. Levels of circulating betatrophin/ANGPTL8 were significantly increased exclusively in septic patients with concurrent hypertriacylglyceridemia > 1.7 mmol/l (“yes”, n = 7) compared to septic subjects and controls with absent dyslipidemia (“no”, n = 6 and n = 15, respectively; *p* = 0.017 and *p* = 0.0015 after post hoc correction, overall significance according to Kruskal–Wallis *p* = 0.0025). No significant effects became apparent in the insulin-resistance group (*p* > 0.05; “yes”, n = 21; “no”, n = 11). IR, insulin resistance. * *p* < 0.05, ** *p* < 0.01 in Kruskal-Wallis test and post-hoc Dunn correction.

We found a correlation between betatrophin/ANGPTL8 and levels of circulating TAG (*p* < 0.05; Figure 3). Therefore, we further stratified betatrophin/ANGPTL8 according to hypertriacylglyceridemia as classified by recent guidelines of the European Society of Cardiology [24]. Thereby, increased circulating betatrophin/ANGPTL8 concentrations were traceable in septic patients with concurring hypertriacylglyceridemia (*p* < 0.05; Figure 4). In contrast, circulating betatrophin/ANGPTL8 showed no correlation with any of the other studied lipid classes, i.e., LDL (r = 0.019; *p* = 0.95), HDL (r = 0.033; *p* = 0.914), TC (r = −0.46; *p* = 0.12), and FFA (r = 0.55; *p* = 0.055). The same remains true for betatrophin/ANGPTL8 mRNA expression in VAT (*p* > 0.05, respectively; data not shown).

For levels of circulating betatrophin/ANGPTL8, we observed no relationship with the main indicators of inflammation, i.e., CRP (r = 0.032; *p* = 0.91), yet a negative association between betatrophin/ANGPTL8 mRNA levels in VAT and circulating CRP was detectable (r = −0.66; *p* = 0.016).

## 4. Discussion

We provide novel findings showing upregulated circulating betatrophin/ANGPTL8 in septic hypertriacylglyceridemia in the absence of concurrent adaptation of VAT expression.

The potential role of lipid metabolism in sepsis has gained increasing attention in recent years [25,26]. Pathogen-associated lipids, such as LPS from Gram-negative and lipoteichoic acid from Gram-positive bacterial cell membranes, are thought to represent important drivers in the progression from uncomplicated infection to sepsis. LDL, very-low-density lipoprotein (VLDL), and chylomicrons are hypothesized to function as neutralization reservoirs for pathogen-associated lipids with consecutive clearance from the bloodstream by the liver and AT, augmenting a reduction of the pro-inflammatory response [7,25]. Recent data also describe the pivotal role of lipoproteins as a source of protective lipid mediators maintaining endothelial barrier function, as well as another role of lipoproteins in the host response [27,28]. Therefore, a better understanding of lipid metabolism under conditions of systemic bacterial inflammation is thought to potentially offer new treatment strategies.

Subjects of our sepsis group evidently showed signs of organ dysfunction, while at the same time, meeting the criteria of septic dyslipidemia. All our samples were obtained after a fasting period and therefore it is reasonable to assume that TAG were largely composed of VLDL [24]. VLDL levels increase due to reduced clearance and metabolic conversion under conditions of sepsis, and it is recognized that reduced lipoprotein lipase activity is a major contributor to this condition [7]. Remarkably, recent reports suggest betatrophin/ANGPTL8 as an important player regarding lipoprotein lipase inhibition and thereby the reduction of TAG clearance [14,29]. Under septic conditions, the latter adaptation could hypothetically augment the neutralization of inflammation-enhancing pathogen-related lipids. Therefore, our finding of significantly upregulated circulating betatrophin/ANGPTL8 could be interpreted as a compensatory mechanism, contributing to septic hypertriacylglyceridemia along with raised VLDL, in order to ameliorate the inflammatory response to bacterial inflammation.

Interestingly, from the perspective of cardiovascular disease prevention, it had recently been discussed to be puzzling that betatrophin/ANGPTL8 has an additional function regarding the uptake of TAG in peripheral tissues, finally promoting lipid storage in AT [29]. Various previous studies including our own have reported associations of circulating betatrophin/ANGPTL8 with plasma lipids and specifically with circulating triglycerides in collectives suffering from T2D and liver steatosis (i.e., [13,30,31]). From the perspective of systemic inflammation, this relationship could be reasonable since the latter mechanism would help to redistribute inflammation-enhancing lipids into AT, thus neutralizing adverse effects. This hypothesis is indirectly supported by findings on proprotein convertase subtilisin/kexin type 9 (PCSK9), which was also found to be increased in sepsis [25]. PCSK9 impairs the clearance of pathogen-associated lipids via the degradation of LDL receptors in hepatocytes and VLDL receptors in adipocytes. The magnitude of the PCSK9 increase correlates with the risk of organ failure, and septic patients carrying loss-of-function variants of the PCSK9 gene show increased survival [8]. Therefore, augmenting the clearance of pathogen-related lipids represents a potent mechanism, which could be capable of affecting clinical endpoints in human sepsis. This further supports the hypothesis of compensatory upregulation of circulatory betatrophin/ANGPTL8 under conditions of human sepsis for the purpose of neutralization of pathogen-associated lipids. The potentially important role in this scenario is underscored by our finding of an isolated correlation of raised betatrophin/ANGPTL8 levels with hypertriacylglyceridemia.

Interestingly, not all of our septic patients with concurring hypertriacylglyceridemia showed elevated circulating betatrophin/ANGPTL8. Rather, we observed two groups of hypertriaclyglyceridemic subjects, with only one cluster of subjects exposing increased betatrophin/ANGPTL8. We found a negative association of circulating betatrophin/ANGPTL8 with HbA1c in our subgroup analyses. Thus, an interference of chronic insulin resistance/dysglycemia and acute inflammation regarding betatrophin/ANGPTL8 regulation can be hypothesized, since we found no significant association with any further laboratory or clinical parameter evaluated in this study that could otherwise explain our observation. In addition, Metformin was recently shown to act as a betatrophin/ANGPTL8 inhibitor under experimental conditions and we observed significantly reduced VAT expression in treated patients [32]. Otherwise, a minority of the subjects in the insulin-resistant subgroup were exclusively under chronic Metformin medication, which was discontinued according to national treatment standards before elective surgery. Moreover, Metformin treatment was without impact on circulating betatrophin/ANGPLT8 as evidenced by increased circulating levels in the insulin-resistance group as compared to healthy controls. Thus, the results reported herein principally confirm previous data, but remain without significant impact regarding our novel findings on betatrophin/ANGPLT8 under conditions of septic dyslipidemia.

In humans, betatrophin/ANGPTL8 is mainly expressed in the liver and AT [13,29]. In the present study, mRNA expression in VAT correlated with circulating levels in both of our control groups, but not under conditions of sepsis. Therefore, it can be further assumed that proinflammatory events interfere with expressional regulation in AT. This hypothesis is principally supported by the observed negative correlation of circulating CRP with VAT expression in our study. Otherwise, the determination of the expression rate in hepatic tissue was beyond the scope of this study, thus we are unable to state, whether this organ contributed to elevated betatrophin/ANGPTL8. Alternatively, betatrophin/ANGPTL8 degradation could be reduced under conditions of systemic inflammation, which was, however, also not the subject of our study protocol.

A major limiting factor is the observational nature of this explorative study, and we can therefore not provide evidence for mechanisms or causal relationships. Additionally, as we included consecutive patients undergoing abdominal surgery, sample sizes are significantly different between groups. In addition, sample sizes were small, especially in the sepsis group, as we aimed to characterize the AT expression of betatrophin/ANGPTL8, and thus were restricted to septic patients undergoing open abdominal surgery. Therefore, we emphasize the explorative nature of our findings in this group, which need confirmation by future research. Furthermore, our results could have been influenced by a potential selection bias. Insulin-resistant and septic patients had a higher mean BMI, were older, and included a higher percentage of males, potentially contributing to the observed findings. Otherwise, particularly insulin-resistant and septic patients were comparable in that regard, and after stratification, we found a significant increase in betatrophin/ANGPTL8 in subjects with concurring sepsis and hypertriacylglyceridemia. Furthermore, metabolically healthy controls with absent systemic inflammation and patients with peritoneal sepsis showed comparable HbA1c, but significantly different circulating betatrophin/ANGPTL8, suggesting that chronic derangements of glucose metabolism or insulin resistance have not been the major driver of our findings in sepsis. Moreover, a recent review provided evidence of conflicting metabolic functions of specific adipokines, i.e., adiponectin, chemerin, and betatrophin/ANGPTL8 [33]. However, the evaluation of such effects was beyond the scope of our study protocol, and we can therefore not state whether this could have contributed to the presented results.

Finally, although we did not use etomidate for anesthesia induction due to its potentially mortality-increasing effects in septic subjects, a propofol bolus was injected instead [34]. Propofol is a lipid emulsion providing triacylgylcerides from soy oil. Since blood samples were taken after surgery in the sepsis group, propofol could theoretically have influenced circulating triacylgylceride levels and accordingly betatrophin/ANGPTL8 in an indirect manner. Moreover, propofol could hypothetically have influenced measures of insulin resistance in the sepsis group [35]. Otherwise, it is not very probable that a single bolus of propofol was sufficient to modulate, i.e., HOMA-IR or circulating triacylglycerides, since a maximum of one or two grams of metabolizable soy lipids were applied with anesthesia induction.

Due to the lack of data and the low number of studied subjects, we cannot provide results on clinical outcomes, which should be addressed in independent studies. In addition, we studied betatrophin/ANGPTL8 only at one single time point during the early acute phase of sepsis, thus we are unable to provide a trajectory during the course of the disease. Lastly, our data are exclusively representative of patients suffering from peritoneal sepsis with a Caucasian background and are not generalizable to other ethnicities or septic entities.

## 5. Conclusions

We provide the first evidence on the regulation of betatrophin/ANGPTL8 under conditions of septic dyslipidemia. We found an upregulation of circulating betatrophin/ANGPTL8 in subjects with septic hypertriacylglyceridemia.

## Figures and Tables

**Table 1:** Baseline characteristics of study patients.

Variable	Control	Insulin Resistance	Sepsis	*p*-Value
Group (% male)	18 (33)	32 (69)	16 (56)	0.053 *
Age [a]	56 ± 11	65 ± 7	70 ± 9	<0.001
BMI [kg/m^2^]	24.9 ± 4.1	28.4 ± 4.4	29.1 ± 4.4	0.007
HOMA-IR [AU]	1.5 ± 1.0	4.1 ± 5.1	4.3 ± 4.8	0.080
Fasting Glucose [mmol/L]	5.2 ± 0.4	7.6 ± 2.5	5.8 ± 1.8	<0.001
HbA1c [%]	5.5 ± 0.4	7.0 ± 1.7	5.9 ± 1.1	<0.001
HDL [mmol/L]	1.5 ± 0.46	1.13 ± 0.3	0.47 ± 0.88	<0.001
LDL [mmol/L]	3.25 ± 0.90	3.41 ± 1.15	0.74 ± 0.57	<0.001
TC [mmol/L]	5.06 ± 0.98	5.09 ± 1.27	2.35 ± 0.85	<0.001
TAG [mmol/L]	1.48 ± 1.09	2.13 ± 0.88	2.21 ± 1.01	0.003
ALAT [mU/L]	0.56 ± 0.17	0.64 ± 0.29	0.88 ± 0.73	0.695
GGT [mU/L]	0.90 ± 0.62	1.61 ± 2.56	2.71 ± 2.41	0.047
CRP [mg/dL]	3.8 ± 2.5	12.4 ± 32.3	221.2 ± 99.6	<0.001
Leukocytes [Gpt/mL]	6.0 ± 1.5	7.1 ± 1.9	16.0 ± 6.1	<0.001
Platelets [Gpt/mL]	236 ± 68	256 ± 71	330 ± 176	0.175
Thromboplastine time [%]	109 ± 11	101 ± 17	76 ± 10	<0.001
Creatinine [mmol/L]	67 ± 11	89 ± 25	189 ± 116	<0.001
Lipid lowering drugs [%]	0	16	6	-

* χ^2^ test.

## Data Availability

The datasets used and/or analyzed during the current study are available from the corresponding author on reasonable request.

## Supplementary Materials

Available online at: https://www.mdpi.com/article/10.3390/biomedicines10123151/s1. Supplementary Table S1: Indications for open abdominal surgery, Supplementary Table S2: Parameters of disease severity in septic patients with detectable circulating betatrophin, Supplementary Figure S1: Subgroup related associations of parameters of glucose metabolism with betatrophin/ANGPTL8 and Metformin treatment.

## Abbreviations

ACTB: β-Actin; ALAT, alanine aminotransferase; ANGPTL8, angiopoietin-like protein 8; AT, adipose tissue; BMI, Body Mass Index; CRP, C-reactive protein; FFA, free fatty acids; GAPDH, glyceraldehyde-3-phosphate dehydrogenase; GGT, γ-glutamyl transferase; HbA1c, glycated hemoglobin A1c; HDL, high-density lipoprotein; HMBS, hydroxymethylbilan synthase; HOMA-IR, homeostasis model assessment of insulin resistance; HPRT, hypoxanthin-phosphoribosyltransferase; LDL, low-density lipoprotein; LPS, lipopolysaccharide; PCSK9, proprotein convertase subtilisin/kexin type 9; TAG, triacylglycerides; TC, total cholesterol; T2D, type 2 diabetes mellitus; VAT, visceral adipose tissue; VLDL, very -low-density lipoprotein
