# Supplementary material for: Associations of Betatrophin/ANGPTL8 with Septic Dyslipidemia in Human Peritonitis: An Explorative Analysis
Source: Biomedicines. 2022 Dec 6;10(12):3151. doi: 10.3390/biomedicines10123151 (PMC9775570; doi:10.3390/biomedicines10123151)
Supplement: Supplementary file 1 [file biomedicines-10-03151-s001.zip › biomedicines-2017139-supplementary.pdf]

Suppl. Table S1: Indications for open abdominal surgery

	Control	Insulin resistance	Sepsis
**Liver resection**			
benign liver disease	**4**	**2**	**-**
primary hepatic or biliary malignancy	**1**	**9**	**-**
secondary hepatic malignancy	**7**	**9**	**-**
**Gastrointestinal (GI) surgery**			
benign GI disease	**2**	**3**	**-**
GI malignancy	**3**	**5**	**-**
**Pancreatic surgery**			
Pancreatic carcinoma	**1**	**3**	**-**
**Other**			
Splenic metastasis	**0**	**1**	**-**
**Septic operations**			
Insufficiency of GI anastomosis	**-**	**-**	**3**
GI perforation	**-**	**-**	**4**
GI ischaemia	**-**	**-**	**1**
Insufficiency of pancreatic anastomosis	**-**	**-**	**3**
Necrotizing pancreatitis	**-**	**-**	**1**
Abscess or fistula	**-**	**-**	**4**

Suppl. Table S2: Parameters of disease severity in septic patients with detectable circulating betatrophin.

ICU stay [d]	11.2 ± 10.1
28 day mortality [% of total]	[33]
Baseline APACHE II	21.5 ± 6.5
Baseline SOFA	9.1 ± 3.3
Microbiological culture positivity [% of total]	40
New dialysis treatment ICU [% of total]	47
Vasopressor support on ICU [% of total]	80
Norepinephrine [%]	80
Highest Norepinephrine dose during ICU stay [µg/kg/min.]	0.5 ± 0.4
Invasive ventilation during ICU stay [% of total]	73
Highest FiO during ICU stay	0.6 ± 0.2
Highest PEEP during ICU stay	8.7 ± 3.0
Highest lactate during ICU stay [mmol/L]	4.6 ± 3.7
Highest blood glucose during ICU stay [mmol/L]	9.9 ± 3.7
Lowest blood glucose during ICU stay [mmol/L]	4.0 ± 0.9
Enteral feeding during ICU stay [% of total]	80
Parenteral feeding during ICU stay [% of total]	0
Preexisting diabetes mellitus [n], insulin dependent (% of total)	6 (40)

Data are shown as Mean ± SD

Suppl. Figure S1: Subgroup related associations of parameters of glucose metabolism with betatrophin/ANGPTL8 and Metformin treatment.

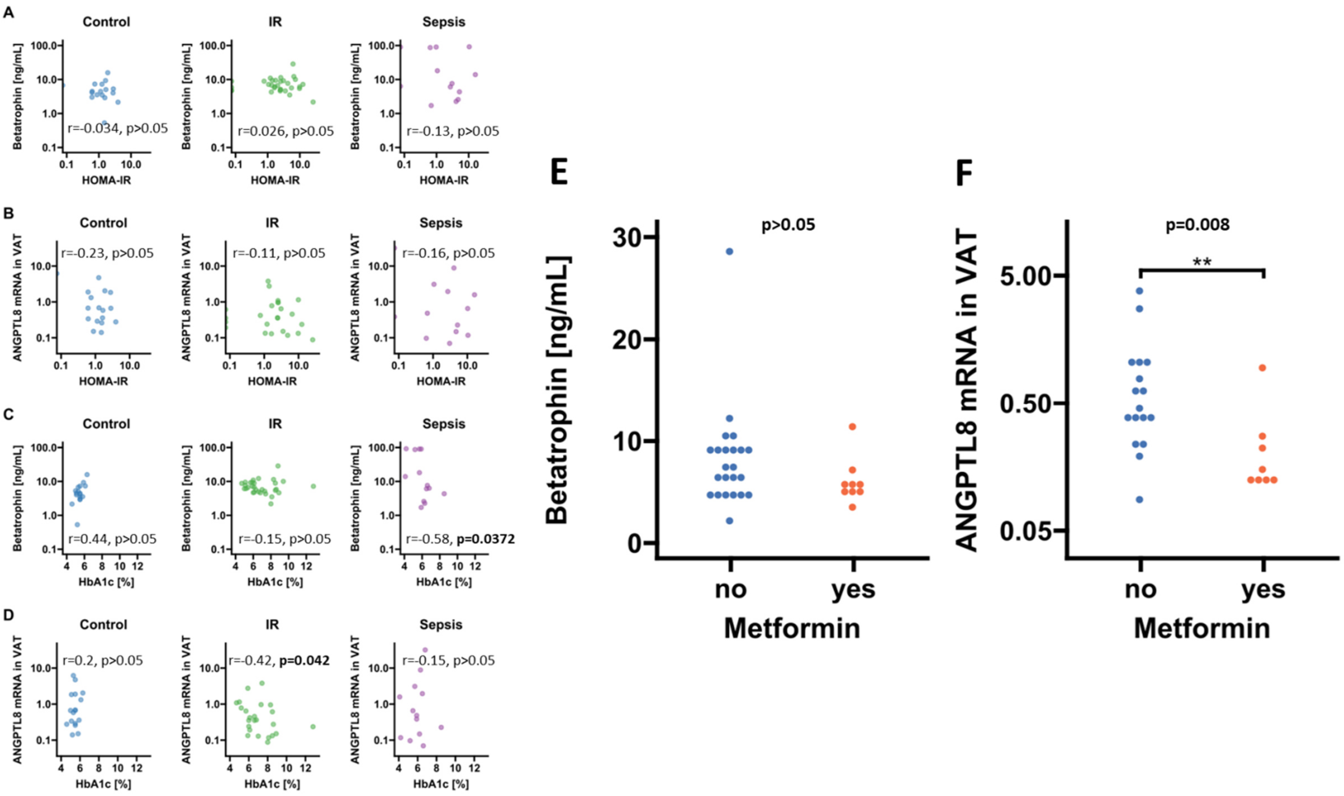

1A-D: Subgroup related correlation analyses of betatrophin/ANGPTL8 mRNA expression in VAT and circulating levels with HOMA-IR and HbA1c are shown. Significant associations are shown in bold. However, after adjustment for multiple testing the observed relationships remained not longer significant.

1E-D: Circulating betatrophin/ANGPTL8 (E) and mRNA expression in VAT are shown in subjects with chronic preoperative Metformin treatment vs. study participants without treatment. According to national diabetes treatment standards metformin was discontinued before elective surgery. All subjects treated with metformin were patients suffering from advanced type 2 diabetes.

HbA1c, glycated hemoglobin 1Ac; VAT, visceral adipose tissue.
